# P-671. Changing Epidemiology of RSV and Associated Hospitalizations in U.S. Children: A Retrospective Analysis (2016-2023)

**DOI:** 10.1093/ofid/ofaf695.884

**Published:** 2026-01-11

**Authors:** Nirma Khatri Vadlamudi, Julia Yang, Rachel Reise

**Affiliations:** University of Florida, Gainesville, FL; University of Florida, Gainesville, FL; University of Florida, College of Pharmacy, Gainesville, Florida

## Abstract

**Background:**

Respiratory syncytial virus (RSV) is the predominant cause of bronchiolitis and pneumonia in pediatric populations, particularly among infants under 12 months of age. Non-pharmaceutical interventions (NPIs) implemented during the COVID-19 pandemic significantly reduced RSV prevalence. Following the relaxation of preventive measures, RSV infections resurged markedly in 2022-2023, resulting in the overwhelming of healthcare systems across North America. This investigation aimed to characterize temporal trends and epidemiological shifts in RSV-associated hospitalizations among U.S. children aged < 17 from 2016 to2023, with particular emphasis on age distribution patterns during pre-pandemic and post-pandemic eras.

**Methods:**

We conducted a retrospective analysis of RSV-associated healthcare utilization using the Merative MarketScan® Commercial Claims and Encounters Database spanning 2016-2023, with a cohort comprising pediatric subjects aged 0-17 years. RSV-attributable episodes were captured using ICD-10-CM diagnostic codes (B97.4, J12.1, J20.5, J21.0, J21.8, J21.9). We considered multiple encounters within 30 days as part of the same episode. Annual incidence rates of RSV hospitalization were calculated and stratified by age cohorts (< 1, 1, 2–4, and 5–17 years). Temporal analysis between the pre-COVID-19 pandemic (2016-2019) and post-pandemic (2021-2023) periods was conducted using negative binomial regression modeling to quantify changes in RSV hospitalization incidence while controlling for person-years.

**Results:**

Over eight years, our study identified 48,386 RSV-associated hospitalizations in the U.S. from a total follow-up of 39,778,977 person-years. In the post-pandemic era, the overall incidence of RSV-associated hospitalizations among children of all ages did not differ significantly from the pre-pandemic period (IRR: 1.22; 95% CI: 0.48–3.24). In contrast, a significant increase was observed among children aged 2–4 years (IRR: 1.70; 95% CI: 1.28–2.27).

**Conclusion:**

A significant increase in RSV hospitalizations among children aged 2–4 years in the United States underscores the critical need for ongoing surveillance and prevention strategies.

**Disclosures:**

Nirma Khatri Vadlamudi, PhD, MPH, Sanofi: Advisor/Consultant|Sanofi: Honoraria

